# Childhood traumatic experiences and mental health problems in sexually offending and non-sexually offending juveniles

**DOI:** 10.1186/s13034-016-0127-2

**Published:** 2016-11-01

**Authors:** Cyril Boonmann, Thomas Grisso, Laura S. Guy, Olivier F. Colins, Eva A. Mulder, P. Vahl, Lucres M. C. Jansen, Theo A. H. Doreleijers, Robert R. J. M. Vermeiren

**Affiliations:** 1 Department of Child and Adolescent Psychiatry and the EMGO Institute for Health and Care Research, VU University Medical, P.O. Box 303, 1115 ZG Duivendrecht, Amsterdam, The Netherlands; 2 Department of Forensic Child and Adolescent Psychiatry, Psychiatric University Hospitals Basel, Basel, Switzerland; 3 Department of Psychiatry, University of Massachusetts Medical School, Worcester, MA USA; 4 Department of Psychology, Simon Fraser University, Burnaby, BC Canada; 5 Curium-LUMC, Leiden University Medical Center and Academic Workplace Forensic Care for Youth, Leiden, The Netherlands

**Keywords:** Sexual offending juveniles, Childhood sexual abuse, Mental health problems, MAYSI-2

## Abstract

**Objective:**

To examine the relationship between a history of childhood abuse and mental health problems in juveniles who sexually offended (JSOs) over and above general offending behavior.

**Methods:**

A sample of 44 JSOs incarcerated in two juvenile detention centers in the Netherlands between May 2008 and March 2014 were examined for childhood abuse history (Childhood Trauma Questionnaire-Short Form) and mental health problems (Massachusetts Youth Screening Instrument-Version 2). Furthermore, the connection between childhood abuse and mental health problems in JSOs was compared to a sample of 44 propensity score matched juveniles who offended non-sexually (non-JSOs).

**Results:**

In JSOs, sexual abuse was related to anger problems, suicidal ideation, and thought disturbance. These associations were significantly stronger in JSOs than in non-JSOs.

**Conclusions:**

Our results suggest that the relationship between childhood abuse and both internalizing and externalizing mental health problems is of more salience for understanding sexual offending than non-sexual offending, and should, therefore, be an important focus in the assessment and treatment of JSOs.

## Background

Childhood traumatic experiences are a major societal problem, with detrimental consequences for the victim. There is clear evidence that childhood abuse is related to an increased prevalence of mental health problems (e.g., [1–3]). Moreover, childhood abuse is a risk factor for later offending behavior [4]. Although childhood abuse is highly prevalent in juveniles who have sexually offended (JSOs) (e.g., [5]), little attention has been devoted to the direct relation between childhood abuse and mental health problems in this specific group of offenders. More insight into this relationship could be of great importance for the assessment and treatment of JSOs.

Previous studies showed that childhood abuse is highly prevalent in JSOs. Based on information reported in Seto and Lalumière’s meta-analysis [5], the mean prevalence rate for sexual abuse in JSOs was 36.9%,^1^ 42.2% for physical abuse, and 48.1% for emotional abuse/neglect. Moreover, JSOs experienced sexual abuse (*d* = 0.62), physical abuse (*d* = 0.19), and emotional abuse/neglect (*d* = 0.28) more often than juveniles who offended non-sexually (non-JSOs) [5].

One hypothesis to explain the higher prevalence of sexual abuse among JSOs compared to non-JSOs is the sexually abused sexual abuser hypothesis (for detailed information see: [5, 6]). According to this hypothesis, juveniles with a history of sexual abuse are at increased risk to engage in sexual offending behavior. Meta-analyses of both adult and juvenile sex offender samples found support for this hypothesis, as sexual abuse histories were relatively more prevalent in offenders who had committed a sex offense than among those who had not [5, 6].

Several explanations have been discussed for the relationship between sexual victimization and later sexual offending. First, sexual abuse victims may be at increased risk for sexual offending vis-à-vis learning (e.g., modeling of their abuser’s behavior) and adoption of positive attitude and beliefs towards sexual behavior between children and adults [7]. Second, sexual abuse may contribute to abnormal or deviant psychosexual development, which in turn may increase risk for sexual offending behavior [6]. Third, the relationship between sexual abuse and sexual offending behavior could be caused indirectly through other third variables, such as mental health problems [6].

With regard to this latter explanation, childhood abuse is related to various mental health problems, including substance abuse, depression, suicidal ideation, anxiety and Post-Traumatic Stress Disorder [8–12]. Because research suggests that a history of childhood abuse is more prevalent among JSOs than non-JSOs [5, 13], one might expect JSOs to have more mental health problems than non-JSOs. In general, JSOs report more internalizing problems (social isolation, anxiety, low self-esteem, thought disturbance) and atypical sexual interests, but fewer externalizing problems, including substance abuse problems, than non-JSOs [5, 13–15]. Hence, it could be hypothesized that the connection between childhood abuse and mental health problems differs among juveniles with and without a history of perpetrating sex offenses.

The aim of the current study is to examine the relationship between childhood abuse and mental health problems in sexual offending behavior, over and above general offending behavior. To do so, we compared the association between childhood abuse and mental health problems in JSOs and non-JSOs. Based on the extant research literature, we hypothesized that there would be a stronger relationship between childhood abuse, especially sexual abuse, and internalizing mental health problems among youth with a history of sexual offending than among youth whose offending histories did not include sexual offenses.

## Methods

### Participants

The sample included 44 male juveniles who sexually offended (i.e., JSOs) and 44 propensity score matched male juvenile non-sexual offenders (i.e., non-JSOs) incarcerated in two juvenile detention centers in the Netherlands between May 2008 and March 2014. Youth were classified as JSO if their official judicial record showed at least one conviction for a sexual offense (*n* = 17), if at least one index offense was a sexual offense (*n* = 26), or if they reported during the assessment that they ever engaged in sexual behavior against someone else’s will (*n* = 6). Non-JSOs were suspected or convicted of violent (e.g., manslaughter, armed robbery) and/or non-violent (e.g., theft, drug dealing) crimes, but did not have a history of sexual offense perpetration. JSOs and non-JSOs were propensity score matched on age and ethnicity. The age range of the total sample was between 13 and 24 years (33% of the offenders were 18 years or older, and 18% were 19 years or older). The mean age of JSOs and non-JSOs was similar [JSOs: 17.0 (SD = 2.0), non-JSOs: 17.7 (SD = 1.8); *t* = 1.8; *p* = .97], as was the proportion of participants who were native Dutch (JSOs: 40.9%, non-JSOs: 22.7%; χ^2^ = 3.4, *p* = .11).

### Procedure

Assessment was part of a standardized self-report mental health screening procedure in the juvenile detention centers used for clinical purposes. Master students and test assistants with a Master’s degree trained by clinically experienced researchers performed the comprehensive assessments. Juveniles and their parents were informed that all information was also used for scientific research after encryption. The relevant institutional review and scientific boards of the juvenile detention centers approved the study and procedure (for more details, see: [16]).

### Instruments

#### Childhood Trauma Questionnaire-Short Form (CTQ-SF)

The CTQ-SF [17, 18] is a 28-item self-report inventory for juveniles and adults (from age 12 and up) that provide brief, reliable, and valid screening for histories of abuse and neglect [18, 19]. It inquires about five types of maltreatment: (1) emotional abuse (e.g., “I thought that my parents wished I had never been born”), (2) physical abuse (e.g., “People in my family hit me so hard that it left me with bruises or marks”), (3) sexual abuse (e.g., “Someone tried to touch me in a sexual way or tried to make me touch them”), (4) emotional neglect (e.g., “There was someone in my family who helped me feel that I was important or special”) and (5) physical neglect (e.g., “I had to wear dirty clothes”). Three items screen for false-negative trauma reports (e.g., “There was nothing I wanted to change about my family”). Participants are asked to rate whether each item is (1) never, (2) rarely, (3) sometimes, (4) often, or (5) very often true. In the Dutch translation [20], one question about molestation was removed due to low correlation with the sexual abuse subscale and high correlation with physical abuse subscale. Translation of the word “molestation” into Dutch was not linked to sexual abuse per se [21]. Internal consistency of the Dutch CTQ-SF ranged from .89 (emotional abuse) to .95 (sexual abuse), with the exception of physical neglect (.63) [21].

#### Massachusetts Youth Screening Instrument-Version 2 (MAYSI-2)

The MAYSI-2 [22, 23] is a brief screening tool to identify youth who are at immediate risk for suicide and increased mental health and substance use needs. Although the MAYSI-2 has been developed for juveniles between the age of 12 and 17, it has been suggested that it can be used also with older youths as long as the results are interpreted carefully [24]. It is one of the most widely used screening instruments for mental health problems in the United States [22, 23], and has been implemented by the Dutch Ministry of Justice as part of the standardized mental health screening at entry to all juvenile justice detention centers in the Netherlands. Based on factor analyses, the MAYSI-2 contains seven scales: Alcohol/Drug Use, Angry-Irritable, Anxious-Depressed, Somatic Complaints, Suicide Ideation, Thought Disturbance, and Traumatic Experiences [22, 23, 25, 26]. All scales except for the Traumatic Experiences scale have two cut-off points. The *caution* cut-off indicates that the score of the youth may have clinical significance; the *warning* cut-off indicates an exceptionally high score compared to other juveniles in juvenile justice institutions.

The MAYSI-2 has acceptable to good internal consistency for the Alcohol/Drug Use, Angry-Irritable, Anxious-Depressed, Somatic Complaints and Suicide Ideation scales, and poor to acceptable internal consistency for the Thought Disturbance and Traumatic Experiences scale [22, 23, 25, 26]. Good concurrent validity has been demonstrated [23, 26, 28–32].

### Statistical analysis

Data were analyzed using International Business Machines Corporation Statistical Package for Social Sciences, version 19 (IBM SPSS 19). The level of significance was set at .01 in order to account for Type I error inflation due to multiple testing. First, differences in childhood trauma scores and mental health scores between JSOs and non-JSOs were examined using *t*-tests. Second, as our data were not normally distributed, we used Spearman Rho Correlations to examine the relation between childhood abuse and mental health problems in JSOs and general offending juveniles. Third, we compared the strength of the relationship between childhood abuse and mental health problems in JSOs and non-JSOs by calculating the difference between the two independent correlation coefficients using software available from http://quantpsy.org [33]. Although the Fischer *r*-to-*z* transformation is a method usually applied to Pearson correlation coefficients, Myers and Sirois [34] showed that this approach performed best in terms of control of Type I error when compared to other strategies. To interpret the magnitude of the correlation coefficients, we followed Cohen’s [35] benchmark of small (*r* = .10), medium (*r* = .30) and large (*r* = .50).

## Results

In Table 1, the descriptive statistics for the CTQ-SF and the MAYSI-2 are presented separately for JSOs and non-JSOs. On the CTQ-SF, both JSOs and non-JSOs reported the highest mean scores on the emotional neglect scale and the lowest mean scores on the sexual abuse scale. The caution cut-off scores of the MAYSI-2 indicate that problems with thought disturbance, depression and anxiety, and somatic complaints are highly prevalent in JSOs. A high number of non-JSOs manifested depressed anxious problems and alcohol/drug use problems. With respect to warning cut-off scores, a high number of JSOs reported problems with alcohol/drug use and thought disturbance. Alcohol/drug use problems also were highly prevalent in non-JSOs. There were no significant differences between JSOs and non-JSOs in reported traumatic experiences (CTQ-SF) or mental health problems (MAYSI-2) (see Table 1).

**Table 1:** CTQ-SF and MAYSI-2 scores for juvenile offenders with and without histories of perpetrating sexual offences

	JSO			Non-JSO			*t*	*p*
	M (SD)	% *(n)*		M (SD)	% *(n)*			
CTQ-SF								
Emotional abuse	6.6 (2.3)	4.7 (2)		6.3 (2.7)	4.5 (2)		−0.52	0.60
Physical abuse	6.0 (2.8)	4.5 (2)		5.9 (2.4)	6.8 (3)		0.95	0.35
Sexual abuse	5.3 (1.0)	4.5 (2)		5.1 (1.0)	4.5 (2)		0.58	0.56
Emotional neglect	9.7 (4.9)	15.9 (7)		8.7 (4.1)	18.2 (8)		0.49	0.63
Physical neglect	6.6 (2.9)	11.4 (5)		6.3 (2.1)	13.6 (6)		0.62	0.54

		Caution % *(n)*	Warning % (*n*)		Caution % (*n*)	Warning % (*n*)		
MAYSI-2								
Alcohol/drug use	1.2 (2.0)	15.9 (7)	6.8 (3)	1.7 (2.4)	20.5 (9)	13.6 (6)	1.16	0.25
Angry-irritable	1.8 (1.7)	6.8 (3)	0.0 (0)	1.9 (2.2)	15.9 (7)	0.0 (0)	0.32	0.75
Depressed anxious	1.5 (1.7)	27.3 (12)	0.0 (0)	1.5 (1.7)	22.7 (10)	4.5 (2)	−0.19	0.85
Somatic complaints	1.7 (1.4)	27.3 (12)	0.0 (0)	1.4 (1.3)	15.9 (7)	2.3 (1)	−1.08	0.28
Suicide ideation	0.3 (0.9)	9.1 (4)	4.5 (2)	0.2 (0.7)	2.3 (1)	2.3 (1)	−0.97	0.33
Thought disturbance	0.5 (0.8)	38.6 (17)	9.1 (4)	0.2 (0.6)	15.9 (7)	4.5 (2)	−1.97	0.05
Traumatic experiences	1.5 (1.2)			1.8 (1.6)			0.82	0.41

*JSO* juveniles who sexually offended, *non-JSO* juveniles who offended non-sexually. The prevalence of maltreatment was based on the moderate to (very) serious cut-off score of the CTQ-SF

In Table 2, correlations between the scales of the CTQ-SF and the MAYSI-2 are presented for JSOs and non-JSOs. For JSOs, 6 of the 30 correlations were medium or large in magnitude [33], whereas this was the case for only 2 of the 30 correlations for non-JSOs. In JSOs, there were significant and large correlations between sexual abuse and anger problems, suicidal ideation, and thought disturbance, as well as between physical neglect and suicidal ideation. Medium correlations were found for emotional abuse and depressed anxious problems, and the Traumatic Experiences scale of the MAYSI-2. In non-JSOs, medium correlations were found for emotional abuse and the Traumatic Experiences scale of the MAYSI-2, and emotional neglect and suicidal ideation. In comparisons of the differences between the two independent correlations in JSOs and non-JSOs, significantly stronger associations were observed among JSOs for the relationship between sexual abuse and anger problems, suicidal ideation, and thought disturbance.

**Table 2:** Spearman rho correlations between scores on MAYSI-2 and CTQ-SF scales

	MAYSI-2																				
	Alcohol/drug use			Angry-irritable			Depressed/anxious			Somatic complaints			Suicide ideation			Thought disturbance			Traumatic experiences		
	JSO	Non-JSO	*p*	JSO	Non-JSO	*p*	JSO	Non-JSO	*p*	JSO	Non-JSO	*p*	JSO	Non-JSO	*p*	JSO	Non-JSO	*p*	JSO	Non-JSO	*p*
CTQ-SF																					
Emotional abuse	.19	.11	.719	.10	.13	.884	.48*	.19	.139	.35	.08	.202	.29	−.10	.078	.24	−.06	.176	.42*	.41*	.983
Physical abuse	.03	.10	.757	−.06	.03	.687	.33	.15	.389	.14	.07	.745	.21	−.03	.273	.14	−.03	.436	.21	.37	.411
Sexual abuse	.01	−.13	.536	.58*	−.09	.001	.33	.04	.165	.10	−.07	.432	.56*	−.05	.002	.55*	−.07	.002	.20	.01	.371
Emotional neglect	−.02	.09	.627	.08	.22	.504	.23	.09	.520	.22	−.13	.107	.17	.41*	.238	.29	.34	.798	.10	.12	.912
Physical neglect	.14	.34	.344	.19	.18	.963	.30	.20	.628	.27	.03	.267	.51*	.33	.319	.32	.32	.968	.12	.37	.221

*JSO* juveniles who sexually offended; *non-JSO* juveniles who offended non-sexually

* *p* < .01; *p* values are for the test of difference between the two independent correlation coefficients

## Discussion

The aim of the current study was to examine the relationship between childhood abuse and mental health problems in sexual offending behavior, over and above offending behavior in general. We found a stronger relationship between childhood sexual abuse and anger problems, suicidal ideation, and thought disturbance in JSOs than in non-JSOs.

In contrast to previous studies [e.g., 5], we did not observe significant differences in history of childhood abuse and current mental health problems between JSOs and non-JSOs. However, our study only included youths in juvenile detention centers, whereas the meta-analysis of Seto and Lalumière [5] included studies with youths sampled throughout different processing points in the juvenile justice system. It has been assumed that the prevalence of mental health problems escalates with increased penetration into “deeper” levels of the juvenile justice system [36]. Based on prevalence studies of mental health problems in juvenile arrestees [37], juveniles brought to court [38], juveniles forensically assessed at the request of the court [39], and incarcerated juveniles [40], Doreleijers [36] hypothesized that the prevalence of mental health problems in youth increases the “deeper” they go into the juvenile justice system. For example, 90% of the incarcerated juveniles reported at least one mental disorder [40]. With such high prevalence rates, statistically significant differences in mental health problems, as well as histories of childhood abuse, become more difficult to identify.

Moreover, it can be argued that, given the objective of the present study, the absence of significant differences between JSOs and non-JSOs in childhood abuse and mental health problems is an advantage, as the relationship of childhood sexual abuse and mental health problems in JSOs compared to non-JSOs is not biased by pre-existing differences between both groups. In line with our hypothesis, we found a relationship between sexual abuse and internalizing mental health problems (i.e., suicidal ideation and thought disturbance) in JSOs, which we did not find in non-JSOs. In addition, we also observed a relationship between sexual abuse in JSOs and externalizing mental health problems (i.e., angry-irritable problems). These results suggest that there is a stronger relation between the degree of sexual abuse and both internalizing and externalizing mental health symptoms in JSOs than there is in non-JSOs.

With regard to the sexually abused sexual abuser hypothesis, we did not find significant differences in experiences of childhood sexual abuse between JSOs and non-JSOs (in contrast to [5, 6]). However, we did find a stronger relationship between childhood sexual abuse and both internalizing and externalizing mental health problems in JSOs than in non-JSOs, indicating that the link between childhood sexual abuse and sexual antisocial behavior might be influenced by mental health problems.

In addition, the relationship between internalizing mental health problems and sexual offending behavior remains incompletely understood. On one hand, internalizing mental health problems may be the result of previously existing problems with sexuality and/or history of sexual abuse. On the other hand, internalizing mental health problems could manifest as a reaction to perpetration of sexual offenses [41, 42]. Hence, as no conclusions can be drawn regarding the causal relationship between internalizing mental health problems and occurrence of sexual offending behavior, future research should investigate the temporal ordering and related causal nexus of internalizing conditions and sexual offense perpetration.

### Limitations

Findings of this study must be interpreted in the context of some limitations. First, previous research showed that JSOs constitute a heterogeneous group with differences in childhood abuse and mental health problems [43–45]. Especially JSOs with child victims, when compared to JSOs with adolescent/adult victims, show more childhood abuse, especially sexual abuse, and more mental health problems. We did not examine subgroups given that our sample of JSOs constituted only 44 offenders. Second, we did not assess the extent, frequency and duration of childhood abuse, which also might have influenced our results. Third, the juvenile detention centers in the current study only admitted males. Therefore, our results cannot be generalized to female offender populations. The fourth limitation refers to the reliability of the results. The CTQ-SF and the MAYSI-2 are both self-report instruments. Therefore, our results may have been biased due to social desirability (e.g., on one hand it is conceivable that a history of maltreatment is kept a secret because of shame or loyalty to the perpetrator, but on the other hand it can be suggested that a history of maltreatment is over-reported to gain justification and/or compassion for one’s behavior). Furthermore, as youths were told that their answers would be used for clinical purposes and for evaluation of their interventions, the (lack of) confidentiality could have affected our results. Moreover, retrospective recall bias [46] also may have played a role in the over- or under-reporting of perceived maltreatment; it has been suggested that more recent maltreatment is more accurately recalled than more distal maltreatment. In addition, amplification of the negativity of the maltreatment (e.g., recall of own abuse history when charged with perpetration of a sexual offense) also could lead to over-reporting of maltreatment [47]. Fifth, the internal consistency of the MAYSI-2 scales Thought Disturbance and Traumatic Experiences have been found to be poor to acceptable [22, 23, 25, 27]. Although lower consistency may be explained by the broadness of the constructs measured, this should be taken into account when interpreting the results. Sixth, by lowering the level of statistical significance to .01 we reduced the probability of making a Type I error. As a result, however, the probability of making Type II errors increased (and power reduced), which should also be taken into account. Seventh, the current study was cross-sectional and, therefore, causal relationships between childhood abuse and mental health problems could not be established. Longitudinal studies are needed to establish this relationship. Finally, beyond mental health problems, other variables could have influenced the relationship between childhood (sexual) abuse sexual offending behavior, such as genetic predisposition, various family factors, and peer influences.

### Implications

Our results suggest that if a youth with a history of perpetrating a sexual offence reports mental health symptoms, especially internalizing mental health problems such as suicidal ideation and thought disturbance or externalizing mental health problems such as angry-irritable problems, there is stronger reason to suspect these symptoms are related to childhood abuse or neglect, especially sexual abuse, than if a youth without a history of sexual offending reports similar symptoms. As internalizing mental health problems are harder to detect than externalizing mental health problems, it is of great importance to assess both internalizing and externalizing mental health problems in JSOs at entry to juvenile detention centers. Furthermore, as we found a stronger relationship between childhood sexual abuse and both internalizing and externalizing mental health problems in JSOs than in non-JSOs, our results suggest the need for a different focus for treatment of JSOs and non-JSOs. For JSOs, perhaps the treatment needs to focus on dealing with the childhood sexual abuse (e.g., trauma-based therapy) if this is determined to be a key risk factor for future offending for that particular youth. Finally, as there is evidence that the relationship between sexual abuse and sexual offending behavior could be caused indirectly through mental health problems, one aspect of sexual violence risk management among juveniles who have experienced sexual abuse could comprise treatment with a focus on healthy development and behaviors in order to prevent sexual offending behavior.

## Footnotes

1 We calculated the mean prevalence rates using information in Table 7 in Seto and Lalumière [5, p. 546]. First, we converted the reported percentages of experienced sexual abuse into proportions. Second, we multiplied the proportion of experienced sexual abuse with the number of adolescent sex offenders per study (i.e., estimate of the number of adolescent sex offenders who experienced sexual abuse). Third, we computed the total number of adolescent sex offenders and the total of the newly created variable proportion experienced sexual abuse*number of adolescent sex offenders (i.e., estimate of the total number of adolescent sex offenders who experienced sexual abuse). Fourth, we divided the total proportion experienced sexual abuse*number of adolescent sex offenders (i.e., estimate of the total number of adolescent sex offenders who experienced sexual abuse) by the total number of adolescent sex offenders. The mean prevalence rates for physical abuse and emotional abuse/neglect were calculated using the same method.
